# A Multi‐Step Deposition Strategy for β‐SiC Coatings on C_f_/C Composites: Achieving Breakthrough Oxidation Resistance and Mechanical Properties

**DOI:** 10.1002/advs.202517256

**Published:** 2025-11-18

**Authors:** Dou Hu, Bing Liu, Xiaoxuan Li, Jia Sun, Tao Li, Yang Xu, Hejun Li, Qiangang Fu

**Affiliations:** ^1^ Shaanxi Key Laboratory of Fiber Reinforced Light‐Weight Composites Northwestern Polytechnical University Xi'an Shaanxi 710072 P. R. China; ^2^ Department of Data and Systems Engineering The University of Hong Kong Hong Kong 999077 P. R. China; ^3^ Henan Key Laboratory of High‐Performance Carbon Fiber Reinforced Composites Institute of Carbon Matrix Composites Henan Academy of Sciences Zhengzhou 450046 P. R. China

**Keywords:** anti‐oxidation, carbon/carbon composites, CVD, multi‐step deposition, SiC

## Abstract

Current SiC coating technologies for anti‐oxidation C_f_/C composites, such as pack cementation, often rely on high‐temperature in situ reaction with severe mechanical property decay. Chemical vapor deposition (CVD) presents a promising alternative for fabricating anti‐oxidation SiC coatings with long service life and high mechanical property. However, existing CVD approaches suffer from poor crack sealing and rapid oxidation loss, leading to insufficient anti‐oxidation time. Therefore, achieving long‐term anti‐oxidation SiC coatings using CVD remains a crucial challenge. This work presents a straightforward multi‐step deposition strategy for fabricating anti‐oxidation C_f_/C composites with long service life and high mechanical properties. The anti‐oxidation time of β‐SiC coated C_f_/C composites reached 659 h at 1500 °C for the first time (within <1% mass loss), far superior to the previously reported index by CVD (1500 °C, < 30 h), and acquired over 30% increase in bending strength compared to uncoated C_f_/C composites. The inhibition on through‐crack sources by multi‐deposition strategy can utilize the oxidation barrier of self‐derived SiO_2_ and keep the stability of coating/substrate interface, thus achieving superior oxidation resistance. This new breakthrough for anti‐oxidation C_f_/C composites opens doors to a broad range of applications, including thermal fields, engine nozzles, brake discs, chemical combustion chambers, and nuclear first wall materials.

## Graphical abstract

A new multi‐step β‐SiC deposition strategy for anti‐oxidation C_f_/C composites successfully achieves dual‐performance breakthrough of anti‐oxidation property (≈700 h at 1500 °C)and mechanical property (bending strength of 215 MPa and adhesion strength of 9.4 MPa). Reasons for this lie in the inhibition on shadow effect during CVD process, ultrahigh coating density (≈99.6%), effective coating thickness (≈120 µm) and continuous β‐SiC sublayers.

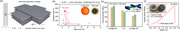

## 1 Introduction

Carbon/carbon (C_f_/C) composites are highly valued for their distinctive combination of properties, including low density (0.9–1.9 g cm^−3^), high specific strength, high specific modulus, high thermal conductivity, low coefficient of thermal expansion (1.0 × 10^−6^ °C), good fracture toughness, and resistance to radiation, wear, and ablation. These attributes make C_f_/C composites valuable materials across various industries, where they find applications in thermal fields for semiconductors,^[^ ^1^, ^2^ ^]^ brake discs for automative,^[^ ^3^ ^]^ engine tail nozzles for aerospace,^[^ ^4^ ^]^ combustion chambers for devices,^[^ ^5^ ^]^ and first wall materials for nuclear energy.^[^ ^6^ ^]^ However, unlocking the full potential of C_f_/C composites hinges on its anti‐oxidation performance at high temperatures.

The SiC‐based coatings are the widely recognized solutions to resist long‐term oxidation under high‐temperature oxidizing atmosphere. Existing SiC coatings with low oxygen permeability, whatever dual‐structure or multi‐component coatings,^[^ ^7^, ^8^, ^9^, ^10^, ^11^ ^]^ are almost based on α‐SiC (hexagonal lattice) coatings from in situ Si reaction methods. The formation of α‐SiC requires a high reaction temperature (Si + C = SiC, >1900 °C) due to low reactivity of Si and C. Currently in situ reacted α‐SiC coatings with a series of modification components can protect C_f_/C composites from oxidation for hundreds of hours, while the strength retention rate is only 40∼70% of original composites. Mechanical damage created during high fabrication temperatures^[^ ^12^ ^]^ can significantly reduce the effectiveness of anti‐oxidation C_f_/C composites. For example, high fabrication temperatures can cause the merging and growth of defects in C_f_/C composites, thus leading to the decrease in bear‐loading capacity and the service stability decline for large‐size or thin‐wall components.^[^ ^13^, ^14^, ^15^ ^]^ While matrix damage control can be relieved through additional procedures such as continuous PyC transition layer^[^ ^16^ ^]^ and porous pre‐coating,^[^ ^17^ ^]^ these processes commonly entail time‐consuming operations, stringent recipes and complicated setups. Moreover, they often have limitations in keeping low‐roughness surface to optimize their utility in applications, thereby suffering from inevitable post‐machining processes and the resulting secondary mechanical damage.^[^ ^18^, ^19^ ^]^

Low fabrication temperatures^[^ ^20^ ^]^ were proved to be beneficial for improving mechanical properties. Chemical vapor deposition (CVD) offers a promising path toward constructing anti‐oxidation SiC coatings with dense structures at low fabrication temperatures (≈1200 °C).^[^ ^21^ ^]^ The gentle atom deposition process will basically not change surface roughness or impair intrinsic structures including carbon fibers and carbon matrix. The β‐SiC (cubic lattice) coating, relying on trichloromethyl silane (CH_3_SiCl_3_) precursors to provide Si and C sources, has been applied on C_f_/C composites to construct low‐roughness and anti‐corrosion surface. However, the one‐step or multi‐step deposition methodologies of β‐SiC coatings for C_f_/C composites have encountered significant limitations in providing long‐term oxidation resistance in the past 20 years. Notably, the maximum reported oxidation protection time for these β‐SiC coatings is less than 30 h at 1500 °C (air atmosphere). Wang et al.^[^ ^22^ ^]^ discussed the deposition parameters of β‐SiC coating including MTS/H_2_ molar ratio, reactor pressure and gas flow rate, which provided comprehensive insights into the regulation of β‐SiC deposition rate and defect density. Qiang et al.^[^ ^23^ ^]^ introduced SiC_NW_ pre‐network to promote the β‐SiC nucleation rate and greatly accelerate its deposition efficiency to form thick β‐SiC coatings by one‐step deposition method. Li et al.^[^ ^24^ ^]^ discussed the reasons for insufficient protection ability of traditional one‐step β‐SiC coating by CVD method and considered the key factors as carbon consumption along the coating/substrate interface caused by penetrating cracks. Compared to one‐step β‐SiC coatings, the SiC_NW_ pre‐network achieved a smaller mass loss rate of β‐SiC coatings but limited short‐term oxidation protection life (17 h, mass loss of 0.8%) at 1500 °C.^[^ ^25^ ^]^ Chen et al.^[^ ^26^ ^]^ also reported the poor anti‐oxidation property of one‐step β‐SiC coatings at 1500 °C and applied laminated [SiC/PyC]_n_ design by multi‐step deposition to prolong the length of penetrating cracks. Despite an improvement in coating toughness, the carbon layers distributed between β‐SiC sub‐layers provided rapid oxygen diffusion path and caused insufficient anti‐oxidation property (10 h, mass loss of 1.3%). Li et al.^[^ ^27^ ^]^ studied the multi‐step deposition process based on multi‐layer [SiC/ZrC]_n_ structures and revealed the formation of carbon‐rich layer on β‐SiC, which resulted from the preferential stacking at the beginning and the end of deposition stage. Overall, there still exists a clear gap in designing high‐performance anti‐oxidation β‐SiC coatings, which is postponed by complex factors including deposition defects, rapid oxidation of C_f_/C substrate, etc. To develop a method to achieve long‐term anti‐oxidation performance of β‐SiC coatings and accordingly provide β‐SiC coating design principles remain a critical goal.

Herein, we proposed a new multi‐step deposition strategy and broke with traditional understanding on anti‐oxidation β‐SiC coating. The multi‐step β‐SiC coating with high strength retention of C_f_/C composites successfully achieved ≈700 h oxidation protection, instead of experiencing complicated steps like various chemical treatment or pre‐introduction of toughening phases.^[^ ^28^ ^]^ This approach draws on the idea of self‐reinforcement^[^ ^29^ ^]^ to obtain β‐SiC coatings with low sensitivity of penetrating cracks. A multi‐step cyclic deposition process enables the successful creation of low roughness surface with cellular‐like grains (≈7.1 ± 0.4 µm) and continuous β‐SiC layer with high crack tolerance to inhibit rapid oxygen diffusion and carbon consumption at the coating/substrate interface, compared to traditional one‐step or multi‐step deposition strategy. These microstructures allow C_f_/C composites to resist long‐term oxygen invasion at 1500 °C and exhibit amazing mechanical property that improves rather than declines. The long‐term protection mechanism is further explained by an oxidized β‐SiC model with distributed cracks and mixed SiO_2_ film, while the superior mechanical properties are revealed via potential fiber protection and surface toughening effects.

## 2 Results and Discussion

### 2.1 Anti‐Oxidation Performance of the Multi‐Step β‐SiC Coatings

**Figure 1:**
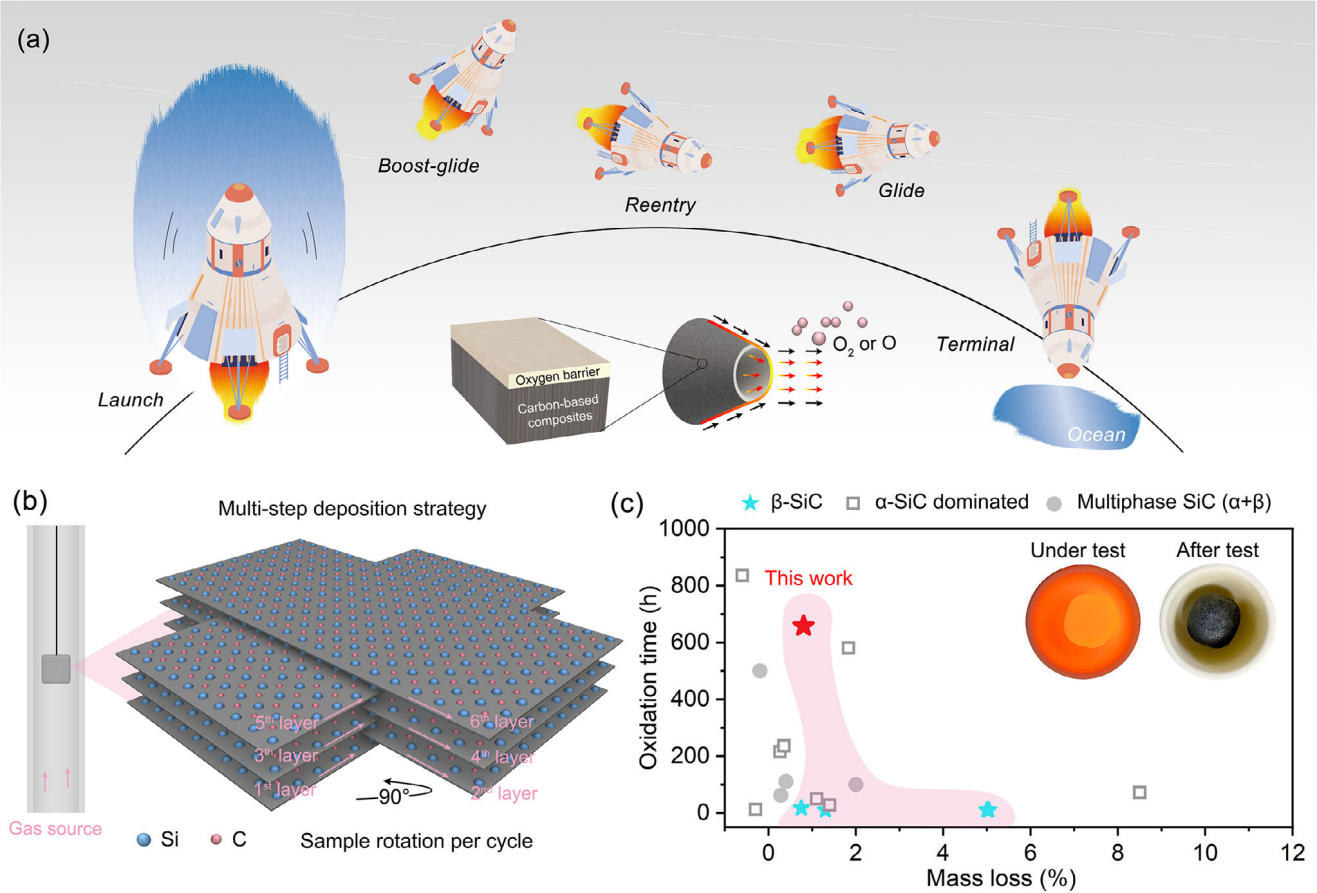
Design idea and anti‐oxidation properties of β‐SiC coated C_f_/C composites: a) Schematic diagram of anti‐oxidation C_f_/C composites for future aircraft engines. b) Multi‐step β‐SiC deposition strategy for C_f_/C composites. c) Comparison of oxidation time of anti‐oxidation C_f_/C composites with previously reported work.^[^ ^11^, ^24^, ^25^, ^26^, ^30^, ^31^, ^32^, ^33^, ^34^, ^35^, ^36^, ^37^, ^38^, ^39^, ^40^, ^41^ ^]^

**Figure** **1**a depicts one of the typical applications of anti‐oxidation C_f_/C composites for aerospace, where C_f_/C substrate mainly contributes to mechanical loading and SiC‐based coatings prevent oxygen‐containing flow invasion. In traditional view, β‐SiC with lower oxygen absorption energy (prepared by CVD) than α‐SiC (from Si in situ reaction) was more difficult to form SiO_2_ oxygen barrier, more inconvenient to seal cracks by additives and harder to control intrinsic penetrating cracks, thus not suitable for providing long‐term oxidation protection in thermal cycling environments. To break the perception, we employed a multi‐step β‐SiC deposition strategy to prolong its oxidation protection time, as depicted in Figure 1b, and therefore potential stress concentration, deposition defects and matrix damage were inhibited compared to traditional in situ reaction process. Figure S1 (Supporting Information) clarifies the efficacy of our multi‐step deposition strategy, which aims to eliminate the shadow effect while obtaining dense and thick β‐SiC coatings. The shadow effect, as depicted in Figure S1a (Supporting Information), commonly results from the discontinuous deposition of reactive gas sources at the hanging areas. Different from typically cellular‐like structure (Figure S1b, Supporting Information), the β‐SiC particles are loosely packed in the shaded areas. From the requirements of anti‐oxidation coatings on the integrity and density, the shadow effect will easily cause fatal oxygen permeation channels.

Figure 1c compares the oxidation time of multi‐step β‐SiC coatings with other β‐SiC (CVD, preparation temperatures of 1000—1500 °C), α‐SiC dominated (in situ reaction, ≈2100 °C) and multiphase SiC (β+α, in situ reaction, 1600—1900 °C) coatings. To acquire long‐term oxidation protection, various additives, pre‐network or alternate design were applied. Table S1 (Supporting Information) summarizes the anti‐oxidation properties of SiC‐based coatings for C_f_/C composites. Notably, the anti‐oxidation β‐SiC coatings by CVD reached the same level to in situ reacted α‐SiC or multiphase SiC coatings for the first time. The inset figures show the macroscopic images of as‐prepared β‐SiC coated samples after 659 h oxidation test at 1500 °C (air atmosphere), which still maintains the coating integrity.

### 2.2 Preparation, Microstructure, and Composition of Multi‐Step β‐SiC Coatings

**Figure 2:**
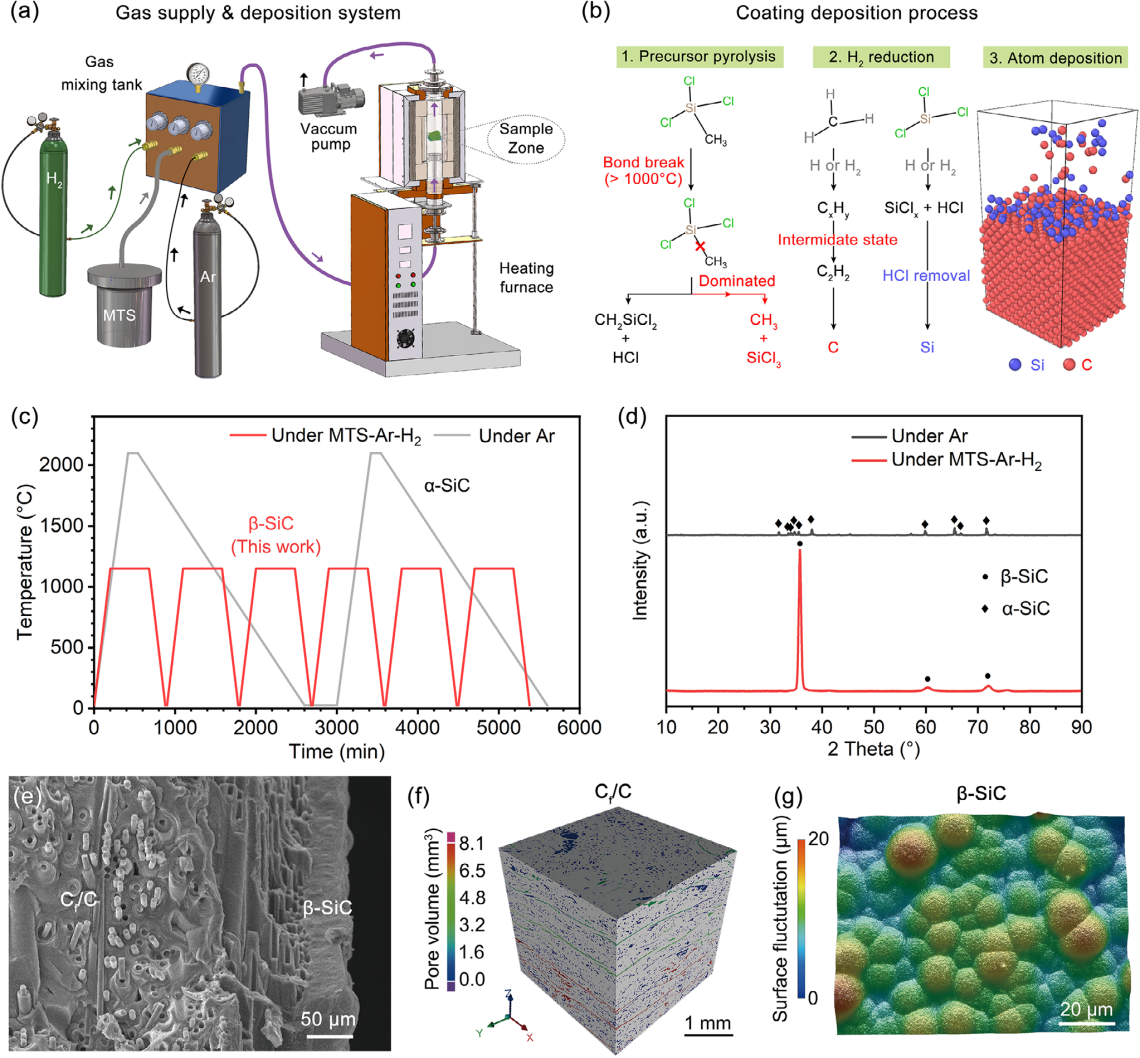
Fabrication process and microstructure analysis of β‐SiC coated C_f_/C composites: a) Schematic diagram of gas supply and deposition device system of β‐SiC coating. b) Deposition mechanism of β‐SiC coating from MTS‐ Ar‐H_2_ precursors. c) Temperature ramping profile of β‐SiC coating under MTS‐ Ar‐H_2_ and α‐SiC coating under Ar for comparison. d) XRD patterns of fabricated α‐SiC and β‐SiC coatings. e) Cross‐section image of β‐SiC coated C_f_/C composites after two deposition cycles. f) Micro‐CT analysis of C_f_/C composites with an observed region of 7 mm × 7 mm × 7 mm; g) Surface morphology of β‐SiC coating.

A multi‐step deposition strategy of β‐SiC coating was developed based on CVD technique, utilizing MTS‐Ar‐H_2_ mixture as gas source, as shown in the schematic diagram of gas supply and deposition system in **Figure** **2**a. Generally, the deposition process of β‐SiC (Figure 2b) depends on several steps including precursor pyrolysis of MTS, H_2_ reduction and atom deposition. To minimize the number of atom deposition defects (Figure S1b, Supporting Information) and destroy their connectivity, a multi‐step deposition strategy was applied to obtain dense and thick β‐SiC coatings, and the sample fixed position was rotated by 90° in the next cycle. In contrast to traditional one‐step deposition strategy, Figure S2 (Supporting Information) further makes a comparison between pre‐network method and multi‐step deposition to construct thick coating. The greater coating thickness is considered beneficial to keeping the stability of coating/substrate interface, which is one of the prerequisites for achieving long‐term oxidation protection. Although pre‐network strategy can significantly accelerate coating deposition efficiency and inhibit the shadow effect while obtaining thick β‐SiC coating, the randomly distributed pore network is difficult to fill and thus sacrifice coating internal density, which has been well verified in the SiC_NW_ reinforced β‐SiC coating system.^[^ ^25^ ^]^ From the macroscopic view, the superiority of our multi‐step deposition strategy directly includes achieving the balance between stability of coating/substrate interface and coating internal density.

Figure 2c depicts the temperature‐time curves of multi‐step β‐SiC deposition and traditional liquid Si in situ reaction strategies. Different from the high fabrication temperatures (1900–2100° C) of α‐SiC coatings, our multi‐step strategy can lower the deposition temperature to 1150 °C at similar deposition times. The mechanical properties of C_f_/C composites mainly result from their internal continuous carbon fibers and pyrolytic carbon (PyC) matrix. On the one hand, high‐temperature in situ reaction process can reduce the tensile strength of carbon fibers, which is attributed to the increased probability of large‐sized micro‐defects in carbon fibers during high‐temperature graphitization process. On the other hand, the low deposition temperature of β‐SiC coating is similar with preparation temperatures of C_f_/C composites, which is beneficial to the maintenance of loading capacity of PyC matrix.^[^ ^15^ ^]^ Figure S3 (Supporting Information) further provides the SEM images of β‐SiC and α‐SiC coatings with different deposition cycles. The deposition thickness of β‐SiC coating reaches up to ≈20–30 µm per cycle according to Figure S3a–c (Supporting Information), while the reactive layer of α‐SiC coating with stacked grains in Figure S3d–f (Supporting Information) exhibits uneven characteristics per cycle, ≈50–100 µm. From perspective of inhibition on oxygen invasion, the coating thickness can affect its potential oxygen diffusion path so that similar coating thickness for comparison were widely adopted. Herein, we selected the coating thickness within the range of 100–200 µm, which was widely reported in the α‐SiC dominated or multi‐phase SiC coating systems (referring to Table S1, Supporting Information). The multi‐step β‐SiC coatings used for oxidation tests were controlled at the thickness of ≈120 µm (Figure S3c, Supporting Information), while that of pack‐cemented α‐SiC coatings were ≈100 µm (Figure S3e, Supporting Information) for similar thickness control.

Figure 2d compares the phase analytical results of formed β‐SiC and α‐SiC coatings. The narrow and sharp diffraction peaks of β‐SiC coating indicate good crystallization of single β‐SiC phase, mainly corresponding to (111) plane of β‐SiC (JCPDS cards No. 00‐29‐1129). HRTEM image in Figure S3g (Supporting Information) further reveals the selective growth orientation. The α‐SiC coating shows numerous diffraction peaks with low intensity, suggesting the polycrystalline growth of α‐SiC and many intrinsic crystal defects. Obvious dislocations in α‐SiC lattice with a (100) interplanar spacing of 0.262 nm can be observed in Figure S3h (Supporting Information). Numerous defects from α‐SiC grain growth and different inhibition methods will cause varying range of oxidation protection life according to Figure 1d. Besides, for the traditional one‐step β‐SiC deposition strategy, numerous penetrating cracks are commonly formed during oxidation process, thus causing rapid carbon consumption at the coating/substrate interface. It seems so difficult to leverage the anti‐oxidation of SiC materials for C/C composites due to stringent fabrication processes and inevitable defects.

The multi‐deposited β‐SiC coating on C/C composites is displayed in Figure 2e and clean interface between coating and substrate can be seen. Micro‐CT analytical results on C/C composites exhibit large interlayer pores (red and green parts) and small in‐plane pores with pore size of <1.6 mm^3^ (blue parts). The selected observed zone is 6.8 × 6.8 × 6.8 mm^3^ and the calculated porosity is 9.6%. To further discuss the relationship between porosity and corresponding surface area to contact oxygen, several theoretical models are established in Figure S4 (Supporting Information). Herein, the open pores are simplified as connected column pores at a total porosity of 10%, and the cubic region is set as 10 × 10 × 10 mm^3^. Their surface area significantly increases with pore size declining. Compared to completely dense structure, 10% porosity will bring over 170 times increase in surface area at the average pore size of 20 µm. From Figure 2g, the β‐SiC coating with smooth surface and invisible defects is composed of nearly spherical particles with a 12.9 ± 2.6 µm diameter. The formation of cellular structure of β‐SiC coating is typically governed by relatively low deposition temperature and gas flowing rate.^[^ ^22^ ^]^ The key premise of heterogeneous nucleation will decide whether continuous β‐SiC coating can form on the substrate. As the temperature increases in β‐SiC deposition process, the kinetic energy and collision frequency of gas molecules will be amplified to promote coating deposition. From the aspect of nucleation rate and growth competition mechanism, high nucleation rate and low growth rate promote the formation of equiaxed crystals, while high growth rate and low nucleation rate accelerate the formation of columnar crystals. The strong (111) preferential orientation in the XRD pattern of as‐deposited β‐SiC coating suggests the continuous epitaxial growth in the cellular structure. From another aspect of reaction kinetics and mass transfer control mechanisms, once the gas reactants need to pass through multiple steps from gaseous precursors to substrate and the reaction rate is limited, the mass transport will promote preferential orientation of a certain crystal plane. Figure S5 (Supporting Information) further presents the roughness of α‐SiC and β‐SiC coated C_f_/C composites. The β‐SiC coatings prepared by CVD exhibit much higher surface quality, the average roughness of which is no more than 1/3 of in situ reacted α‐SiC coatings.

### 2.3 Long‐Term Oxidation Behavior of Multi‐Step β‐SiC Coated Cf/C Composites

Anti‐oxidation coatings for C_f_/C composites leverage a continuous layer with oxygen‐isolation and oxygen‐consuming materials, which generally require the density, stability and low oxygen permeation ability of as‐deposited coating structure. To explore the secret of long‐term oxidation protection ability of multi‐step β‐SiC coating, quantitative analyses were conducted from two aspects of self‐derived oxide film and multi‐deposited coating itself.

**Figure 3:**
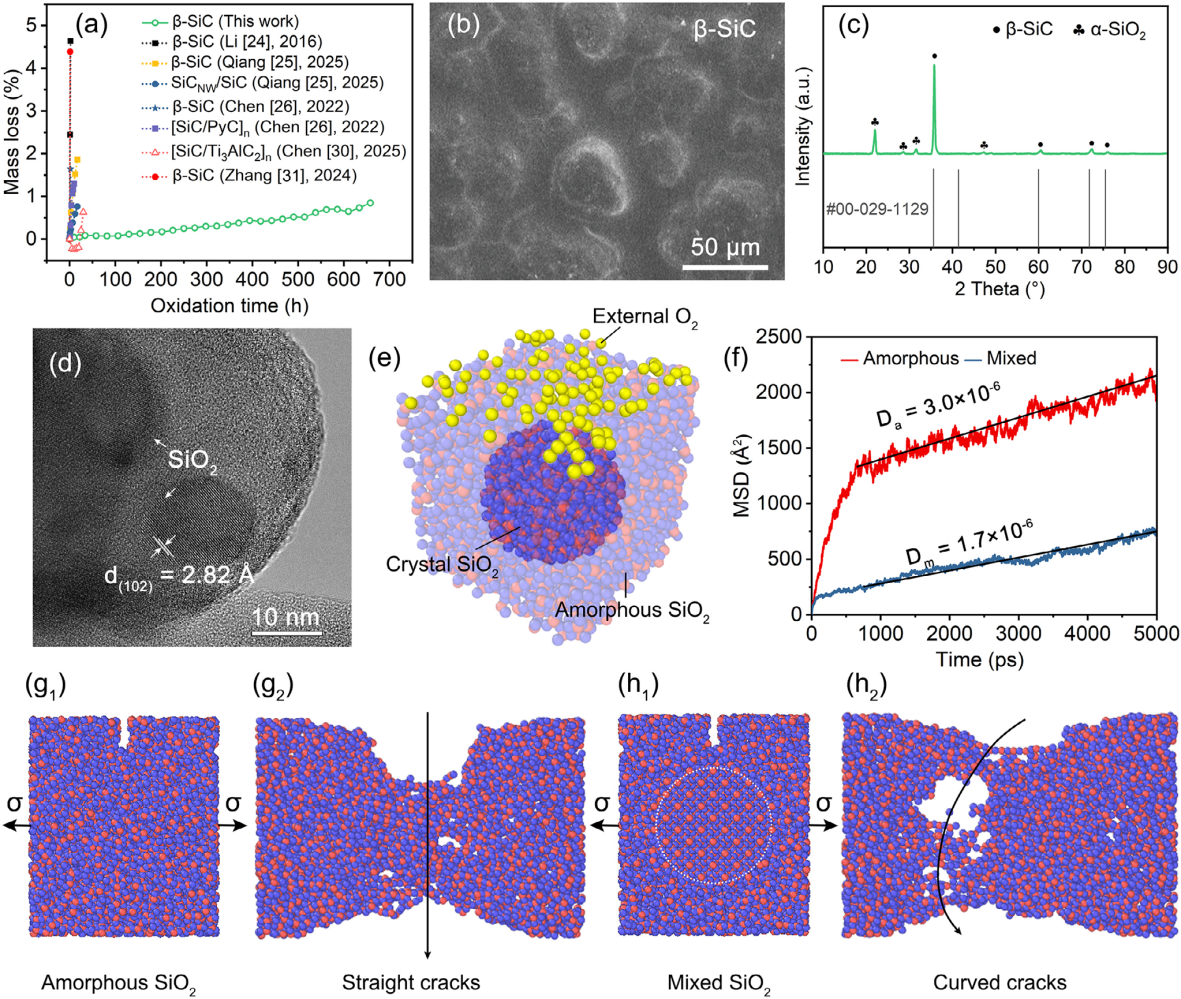
Long‐term oxidation behavior of β‐SiC coating by multi‐step deposition at 1500 °C. a) Weight loss curves of multi‐step β‐SiC and previously reported β‐SiC coatings.^[^ ^24^, ^25^, ^26^, ^30^, ^31^ ^]^ b) Surface SEM image and c) XRD pattern of multi‐step β‐SiC coating oxidized for 659 h. d) TEM characterization and (e) schematic diagram of self‐derived oxide film to resist oxygen invasion. f) Evaluation on oxygen diffusion coefficients of amorphous and mixed SiO_2_ films. Crack propagation in (g_1_‐g_2_) amorphous and (h_1_‐h_2_) mixed SiO_2_ films under tensile stress.

**Figure** **3**a compares the weight loss curves of multi‐step β‐SiC and previously reported β‐SiC coatings at 1500 °C, suggesting the breakthrough oxidation protection ability. A glassy film on spherical particles can be seen on the surface of β‐SiC coating in Figure 3b, and corresponding XRD spectrum (Figure 3c) reveals the formation of crystal SiO_2_. Generally, SiC will be initially oxidized into amorphous SiO_2_ and further precipitate crystal phase by utilizing necessary activation conditions to overcome energy barriers, like external high temperature, internal lattice defects and low viscosity areas. The mixed morphology of amorphous and crystal SiO_2_ film is displayed in Figure 3d, where a series of (102) planes are distributed in amorphous SiO_2_. Generally, the amorphous SiO_2_ film generated by SiC was widely considered as a barrier to control the chemical reaction equilibrium of Si, C and O at the interface of SiC/O_2_. A slow and gradual crystallization process can be helpful to achieve long‐term and stable service at high temperatures.^[^ ^42^ ^43^ ^]^ Once the silica tetrahedral network [SiO_4_]^4−^ is broken by additives or local fluctuation, the crystal SiO_2_ precipitation will occur and reduced its oxygen diffusion rate, due to molecular oxygen can diffuse inward through its internal network voids or exchanging network oxygen.^[^ ^21^ ^]^ The stability of SiO_2_ barrier can be regulated by various doping elements to break/enhance Si‐O network, so its service stability for coating design can be controlled at a wide temperature threshold.

From the perspective of oxygen barrier effect of SiO_2_ film, Figure 3e depicts the schematic diagram of amorphous SiO_2_ pinned by crystal SiO_2_, where the self‐pinning phenomenon from β‐SiC coating is also found beneficial for lowering oxygen diffusion coefficients (D). The mean square displacement (MSD) curves make a contrast between amorphous SiO_2_ and mixed SiO_2_ films according to Figure 3f. The D value of mixed SiO_2_ film (D_m_ = 1.7 × 10^−6^ cm^2^/s) is only about half that of amorphous SiO_2_ film (D_a_ = 3.0 × 10^−6^ cm^2^/s), within the range of experimental measurement results (10^−8^–10^−6^ cm^2^/s) as reported.^[^ ^44^ ^]^ On the other hand, the self‐pinning effect of crystal SiO_2_ exhibits a beneficial effect on crack deflection under thermal stress, as depicted in Figure 3g,h, suggesting the increased crack blocking capacity, in accordance with the crack pinning phenomenon from other mosaic phases in SiO_2_ film.^[^ ^45^ ^]^ This demonstrates that surface film can maintain stability without any element modification in 1500 °C air environment, which meets the necessary enabling factor of stable oxygen barrier for long‐term anti‐oxidation coating design for C_f_/C composites. Figure S6 (Supporting Information) further supplements the dynamic O_2_ diffusion process in mixed and amorphous SiO_2_ films at 1500 °C. The lower MSD of mixed SiO_2_ film can be related to the movement inhibition of central crystal area on various O_2_ molecules. Interestingly, despite the complete SiO_2_ film from β‐SiC coating, the previously accepted view of its worse anti‐oxidation performance suggests that a dense and stable oxide film is merely one of the necessary prerequisites for long‐term anti‐oxidation performance.

**Figure 4:**
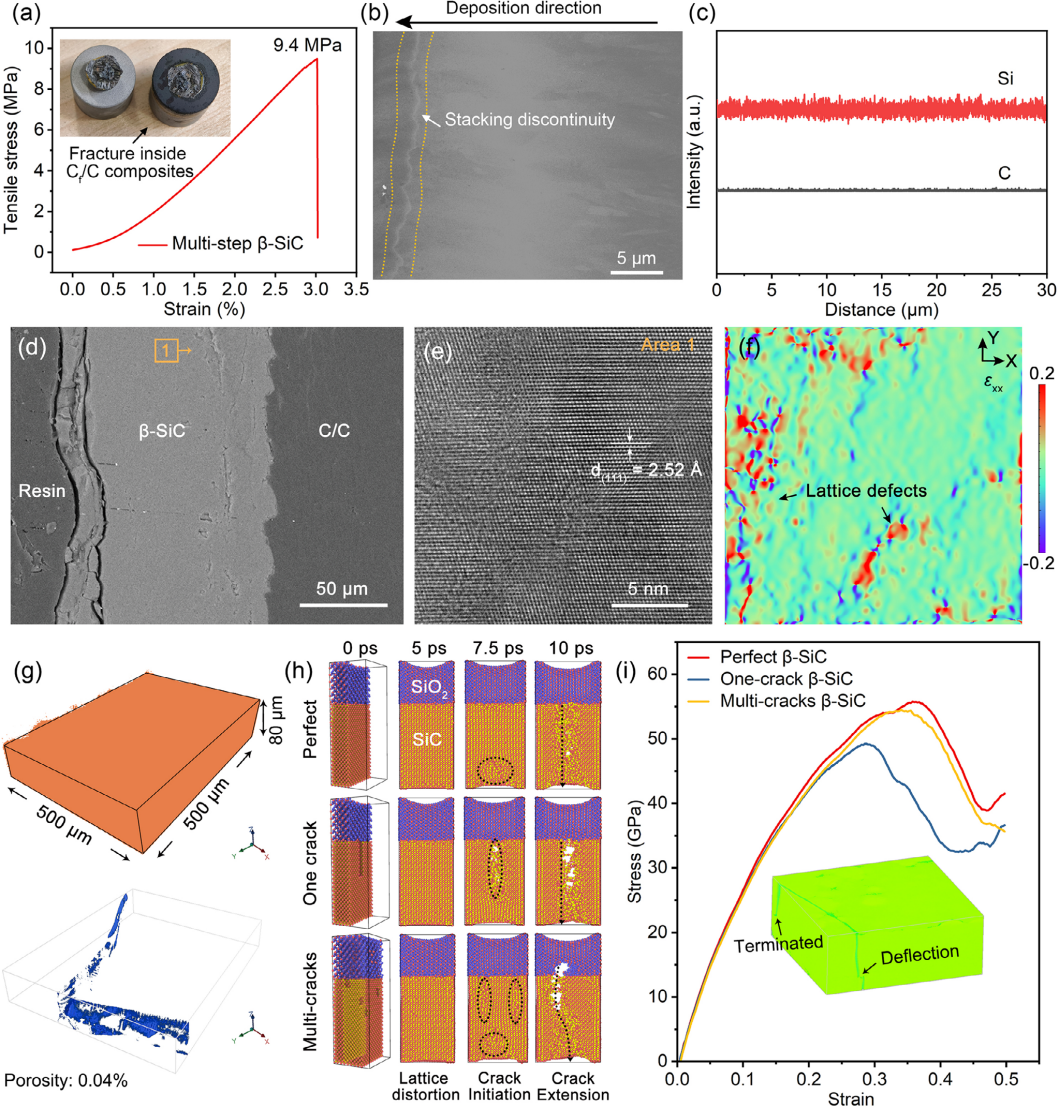
Long‐term oxidation protection mechanism of multi‐step β‐SiC coated C_f_/C composites. a) Pull‐out stress and strain curve with inset fracture photos, where the adhesion strength is measured as more than 9.4 MPa, suggesting the bonding strength between β‐SiC sub‐layers is stronger than that of C_f_/C composites. b) SEM image on the micro‐interfaces of multi‐step β‐SiC coatings, which reveals the ultra‐dense coating structure and stacking discontinuity. c) The line EDS along deposition direction suggests the continuity of β‐SiC component. d) Cross‐section SEM image of multi‐step β‐SiC coating after 659 h oxidation. e) HRTEM image and f) corresponding strain mapping image (ε_xx_) of β‐SiC coating after oxidation. g) Micro‐CT images of β‐SiC coating with an observation region of 500 × 500 × 80 µm to reveal pore distribution. h) Crack propagation process of oxidized β‐SiC coating with different initial crack distribution and i) corresponding stress‐strain curves by MD simulation. The inset figure gives the micro‐CT image of multi‐step β‐SiC coating with an observation region of 300 × 300 × 60 µm to verify the multi‐crack distribution.

From another perspective of multi‐step β‐SiC coating itself, strong adhesion strength at the interface of β‐SiC sub‐layers and at the coating/substrate interface are the prerequisites of inhibiting the formation of rapid oxygen diffusion path. **Figure** **4**a presents the pull‐out stress and strain curve with inset fracture photos, where the adhesion strength is measured as more than 9.4 MPa. The fracture position can be clearly observed inside C_f_/C composites, suggesting that the layer‐by‐layer bonding between sub‐layers of multi‐step β‐SiC coatings is stronger than that of C_f_/C composites. To intuitively display the micro‐interfaces between sub‐layers due to several deposition cycles, the SEM image in Figure 4b reveals the stacking discontinuity in as‐designed β‐SiC coatings, while the corresponding line EDS results (Figure 4c) along deposition direction suggests the continuity of β‐SiC component. Compared to other one‐step or multi‐step deposited coatings as supplemented in Figure S7 (Supporting Information), our multi‐step deposition strategy exhibits the advantage of adhesion strength by process regulation of continuous interfaces between sub‐layers without carbon‐rich layer mutation, only slightly stacking discontinuity.

Figure 4d displays the cross‐section image of multi‐step β‐SiC coating after ≈700 h oxidation at 1500 °C, which shows intact interface between β‐SiC sub‐layers and coating/substrate. Compared to the newly deposited β‐SiC (Figure 1g), various lattice defects can be observed in the oxidized β‐SiC coating according to Figures 4e,f. From the perspective of coating density, Figure 4g analyzes the multi‐step β‐SiC coating by micro‐CT (a porosity accuracy of 1 µm), which exhibits the total porosity of 0.04%. Unfortunately, the porosity distribution in some areas is parallel to the direction of coating thickness, suggesting its promotion effect on the formation of rapid oxygen diffusion path to C_f_/C substrate. That is, when compared to traditional one‐step β‐SiC with a thickness of 30–40 µm, their thin barrier layer and potential shadow effect tend to cause instability at the coating/substrate interface during oxidation process, thus resulting in limited protection life.^[^ ^25^, ^26^ ^]^ Our multi‐step β‐SiC coatings with sufficient thickness (≈120 µm) can theoretically extend the crack propagation path by more than 3 times compared to traditional thin coating (≈40 µm). As for α‐SiC coating obtained by in situ reaction method (Figure S3e, Supporting Information), carbon consumption at the coating/substrate interface is traditionally considered much easier between one‐step β‐SiC and C_f_/C composites, due to more evident crack sealing effect in α‐SiC coatings.^[^ ^29^ ^]^ Accordingly, more bubbles and holes resulting from evaporation of residual Si or sintering aids will be maintained on the surface (Figure S8a, Supporting Information). Although the total porosity of α‐SiC coating is estimated to 2.3%, according to Figures S8b,c (Supporting Information), its defect distribution induced by random α‐SiC grains exhibits scattered characteristics and tortuous pathways in large area, instead of locally penetrating path. Similar tortuous pathways can be also achieved in traditional one‐step β‐SiC coatings with high thickness as pre‐network is pre‐introduced (Figure S2, Supporting Information). However, despite the delayed internal carbon consumption by extended coating thickness (300–400 µm) or internal pathways, insufficient oxidation protection life of one‐step β‐SiC coating was also reported in literature^[^ ^25^ ^]^ due to complex defect distribution and reduced internal density. According to Figure S4 (Supporting Information), only 10% porosity will bring over 170 times increase in surface area of distributed pores in β‐SiC coating (average pore size of 20 µm), compared to completely dense coating. Hence, our multi‐step β‐SiC coatings with dense structure (>99.6%) can greatly reduce oxygen contact and invasion, thus beneficial for maintaining the stability of coating/substrate interface (Figure 4d).

To visually clarify the beneficial effects in β‐SiC from aspect of deposition defect distribution, several crack‐distribution models were established and discussed based on experimental results and MD simulation. As illustrated in Figure S1 (Supporting Information), the inevitable shadow effect will easily cause deposition defects during one‐step deposition process and multi‐step deposition strategy can help eliminate or change potential crack distribution. Figure 4h analyzes the crack propagation process of oxidized β‐SiC coating with different crack distribution. Figure S9 (Supporting Information) further supplements the crack initiation and propagation process at the interface SiO_2_ and SiC, uncovering the causes of interface cracks. The initial one‐crack distribution arouses earlier emerging trend of penetrating cracks in contrast to perfect β‐SiC model, while multi‐cracks promote crack deflection. Figure 4i further displays stress‐strain curves obtained from MD simulation. Compared to perfect β‐SiC model, one‐crack β‐SiC model shows 11.5% decrease in tensile strength, while multi‐crack β‐SiC model only exhibits 2.3% decline under thermal stress. Notably, multi‐crack model improves the energy absorption ability of one‐crack β‐SiC by 9.7% under the same defect content. The inset figure reveals the crack deflection phenomenon inside multi‐step β‐SiC coating, suggesting the potential extension of oxygen diffusion path. Since the interface between SiC and C_f_/C substrate is not rapidly corroded by oxygen, the self‐healing characteristic of SiC will continue to block its continuous invasion. Compared to traditional multi‐step deposition strategy, our multi‐step deposition strategy inhibits the formation of carbon‐rich layer during different deposition cycles.^[^ ^26^ ^]^ Hence, the potential rapid oxygen diffusion channels within multi‐step β‐SiC coating are also avoided, while maintaining the thickness and density of the coating, thus escaping the life limitation caused by internal delamination.^[^ ^26^, ^30^ ^]^ Overall, reasons for long‐term oxidation protection of multi‐step β‐SiC coatings can be attributed to the stability between β‐SiC sublayers and at the coating/substrate interface (Figure 4d), reduced oxygen contact area (Figure 4g) and expanded crack blocking capacity (Figure 4i), benefiting from the more continuous, dense and thick coating structure.

### 2.4 Enhancement Mechanisms for Mechanical Property of Multi‐Step β‐SiC Coated C_f_/C Composites

**Figure 5:**
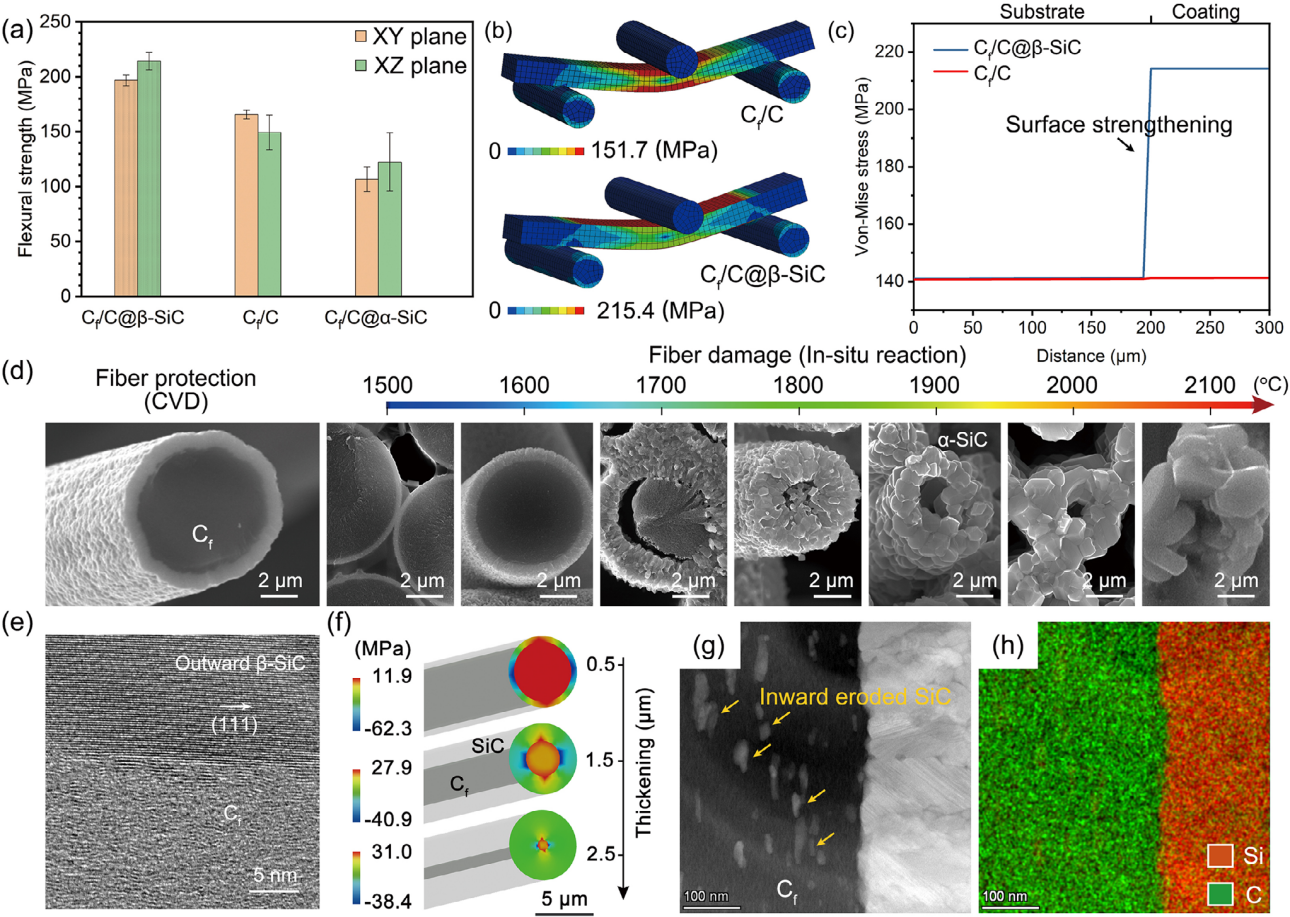
Mechanism analyses of improved mechanical property that outperforms expectations for β‐SiC coated C_f_/C composites. a) Comparison of flexural strength of β‐SiC coated C_f_/C, C_f_/C and α‐SiC coated C_f_/C composites. b) Von‐Mise stress maps under three‐point bending conditions. c) Maximum stress distributions of β‐SiC coated C_f_/C and C_f_/C composites along loading directions.(d) Morphology evolution of carbon fibers during CVD and in situ reaction processes, where the deposition time is controlled at 2 h. Outward deposited β‐SiC and inward eroded α‐SiC respectively exhibit fiber protection and fiber damage. e) Enlarged image at the interface between β‐SiC and C_f_ by CVD. f) Internal stress distribution between SiC and C_f_ with in situ reaction depth increasing. g) Enlarged image at the interface between SiC and C_f_ by in situ reaction method, where the eroded C_f_ at 1500 °C is selected. h) EDS mapping to reveal Si and C distribution corresponding to g).

Mechanical damage of anti‐oxidation C_f_/C composites will show great impact on their applications as load bearing structural components. To further clarify the privilege of β‐SiC coating on anti‐oxidation C_f_/C composites, **Figure** **5**a compares the mechanical performance between C_f_/C composites and their substrate after depositing α‐SiC and multi‐step β‐SiC coatings. On the consideration of thickness ratio (>20:1) between substrate and coating, the bending strength of coated C_f_/C composites is mainly contributed by substrate itself so that the key factor to performance variation lies in how the coating deposition process affects the substrate. Local modification based on the same type of β‐SiC coatings was previously verified little impact on the whole bending strength.^[^ ^46^ ^]^ As mentioned above, our multi‐step deposition strategy has achieved sufficient β‐SiC coating density and thickness, as well as the similarity of multi‐step and one‐step β‐SiC in composition. Despite a small number of stacking discontinuity (Figure 4b), the eliminated negative effects including shadow effect and insufficient density still acquire the breakthrough of oxidation protection life. To visually compare the effects of micro‐interfaces between β‐SiC sub‐layers, Figure S10 (Supporting Information) studied the deformation behavior of β‐SiC coatings with different stacking defects via MD simulation. The increase of lattice defects during β‐SiC deposition will easily cause the transformation from crystal structure to amorphous structure (Figure S10a, Supporting Information), thus leading to a decline of resistance to tensile stress (Figure S10b, Supporting Information). Nevertheless, the deformation discontinuity to inhibit penetrating cracks can be also activated when a small number of stacking defects are distributed at the interfaces (Figure S10c, Supporting Information), in accordance with Figure 4i. Despite up to 10% stacking defects at the interface between β‐SiC sub‐layers, the fracture force almost remains unchanged, suggesting that the micro‐interfaces will not be prone to preferential spallation or cracking under repeated thermal stress.

The multi‐step β‐SiC coated C_f_/C composites by CVD show a performance increase (31.7%–43.2%) compared to C_f_/C composites (149.4 ± 15.8 MPa), while α‐SiC coated C_f_/C composites via in situ reaction exhibit a significant decline (≈35.6%) in bending strength along XY planes. Figure S11a (Supporting Information) supplements the load‐displacement curves of multi‐step β‐SiC and α‐SiC coated samples, which further clarifies the great benefits in mechanical property by applying multi‐step β‐SiC coating technology. Whether for XY or XZ planes of C_f_/C composites, the advantageous effect of multi‐step β‐SiC coating deposition on mechanical properties is that they increase rather than decrease. Reasons for mechanical performance increase induced by β‐SiC coating are mainly attributed to stress re‐distribution effect, as displayed in Figure 5b, which leverage the low porosity advantage of β‐SiC coating and transfer surface stress to denser β‐SiC to inhibit crack initiation trend. Figure S11b (Supporting Information) analyzes the change of open porosity and density after depositing β‐SiC, which further verifies the decrease of surface defects from a macro perspective. Figure 5c shows a sudden increase in stress concentration on the surface of β‐SiC coated samples, suggesting a damage threshold improvement to resist tensile stress.

On the other hand, the mechanical performance evolution of C_f_/C composites affected by anti‐oxidation coating deposition strategy can be specific to fiber damage degree. When not considering in situ reaction between Si melt and carbon fibers, to reduce heat treatment temperatures is widely considered beneficial to inhibit the formation of new internal defects and to keep higher bending strength of C_f_/C composites. Figure 5d displays the effects of β‐SiC or α‐SiC coating deposition process on carbon fibers. The outward β‐SiC layer by CVD is only attached to the surface and exhibits neat interface (Figure 5e), while the in situ reacted SiC layers are gradually embedded into carbon fibers with reaction temperature increasing. Figure S11c (Supporting Information) analyzes the phase evolution of generated SiC layers during in situ reaction process, where the preparation temperatures of α‐SiC coated C_f_/C composites are commonly selected as 1900—2100 °C to form a continuous layer. The aggravated damage above 1700 °C can be further revealed from the evolution of in situ reacted SiC grain size and layer thickness, as shown in Figure S11d (Supporting Information), the increased carbon fiber consumption will damage its load‐bearing capacity and induce stress concentration. Figure 5f further gives the stress distribution of in situ reacted SiC layers into carbon fibers, which indicates the increased stress concentration with reaction layer thickening. Figures 5g,h directly reveal the Si inward diffusion and resulting erosion. That is, reasons for mechanical performance decay encountered by α‐SiC coating technique are mainly due to the inevitable inward erosion that causes fiber load‐bearing capacity to decrease and stress concentration (Figure 5e) to form more crack sources.

Based on above analysis, anti‐oxidation β‐SiC coatings for C_f_/C composites can achieve long‐term high‐temperature protection life without sacrificing their mechanical properties. The dual‐performance breakthrough of anti‐oxidation property and mechanical property can further extend the design principles of anti‐oxidation coatings, including (i) stability of the coating/substrate interface to keep strong interface bonding; (ii) ultra‐dense coating structure with enough thickness to extend oxygen diffusion path and lower oxygen contact area; (iii) stable SiO_2_‐based barrier to ensure long‐term oxygen isolation.

## 3 Conclusion

This work presents a simple and innovative approach for fabricating dense β‐SiC coatings for C_f_/C composites using CVD, where a breakthrough is achieved in long‐term anti‐oxidation performance and superior mechanical properties. We unveil the previously unreported but crucial role of cyclic multi‐step deposition of β‐SiC itself in improving anti‐oxidation performance. The feasibility of applying β‐SiC coatings is successfully demonstrated for long‐service life at high‐temperature oxidizing atmosphere, offering a valuable strategy for future studies. For the traditional one‐step strategy, numerous penetrating cracks are often formed and cause rapid carbon consumption at the coating/substrate interface, due to the inevitable crack sources in shadow areas. Once the through‐crack sources are designed to be inhibited, the resulting oxidation protection time of multi‐step β‐SiC coatings using CVD reach ≈700 h at 1500 °C by leaps and jumps, far superior to previously reported index (< 30 h). MD simulation and experiments reveal that continuous and self‐pinning SiO_2_ films leverage their advantages of stable oxygen barriers, while dense and thick β‐SiC coatings bring out continuous β‐SiC distribution and expanded crack blocking capacity. Remarkably, the bending strength of β‐SiC coated C_f_/C composites increases over 30% than uncoated C_f_/C composites, instead of serious declining up to 35.6% resulting from high‐temperature in situ reaction method. Outward deposited β‐SiC layer effectively avoids inward erosion damage on carbon fibers and internal stress concentration during traditional in situ reaction process at high temperatures. This study opens doors to develop anti‐oxidation C_f_/C composites at low fabrication temperatures and provides guidance for avoiding complicated process steps by appropriate structure designing for broad high‐temperature applications.

## 4 Experimental Section

### Materials

Carbon/carbon (C_f_/C) composites with a density of 1.7 g cm^−3^ were first densified by isothermal chemical vapor infiltration (ICVI) process based on 2.5D needled carbon felt (0.43 g cm^−3^, Yixing Tianniao High Technology Co. Ltd, Jiangsu, China). Methane, nitrogen, argon and hydrogen (99% purity, Xi'an Wei Guang Gas Co., Ltd., China) were purchased as the precursors. The commercial Si, C and Al_2_O_3_ powders (200 mesh) were obtained from Tianjin Fengyue Chemical Co., Ltd., China. Methyltrichlorosilane (MTS, CH_3_SiCl_3_, 99.5%) was purchased from Shandong Xiya Chemical Technology Co. Ltd., China.

### Preparation of α‐SiC Coatings

The Si, C and Al_2_O_3_ powders were mixed uniformly at a mass ratio of 85 wt.%, 10 wt.% and 5 wt.%. The polished and cleaned C_f_/C composites were embedded in mixed powders and then treated at 2100 °C for 2 h in flowing Ar gas. To reduce the intrinsic defects of α‐SiC coatings, the high‐temperature in situ reaction process was repeated twice.

### Preparation of β‐SiC Coatings

The MTS‐Ar‐H_2_ mixed gas was introduced into a tube furnace (Xi'an Jiabo Electric Furnace Co., Ltd., China) and then the deposition temperature was controlled at 1150 °C for 8 h. The MTS weighed ≈150–200 g and the flow rates of Ar and H_2_ were controlled at 100–400 mL min^−1^ and 0.5–1.5 L min^−1^, respectively. To obtain the multi‐step β‐SiC coatings, the procedure was performed for a total of six iterations.

### Material Characterization

The SEM images of α‐SiC and β‐SiC coated C_f_/C composites were obtained using a tungsten filament scanning electron microscope (ZEISS Sigma 300) equipped with an energy dispersive spectroscope (EDS, Oxford INCA, Holland). The phase compositions of the specimens were analyzed by X‐ray diffraction (XRD, X'Pert Pro MPD). The elemental distribution and crystal structure of the specimens were characterized by a transmission electron microscope (TEM, Talos F200X, FEI, USA). Surface roughness was measured by a confocal laser scanning microscope (Optelics C130) with an observed region of 1.25 mm × 1.50 mm. Inner of C_f_/C composites was evaluated by industrial CT (AX2000, German) with an observed region of 7 mm×7 mm×7 mm. Defects of β‐SiC and α‐SiC coatings were detected by X‐CT (ZEISS Xradia 520 Versa) with a maximum resolution of 1 µm.

### Adhesion Test

The bonding strength between the multi‐step β‐SiC coating and C_f_/C substrate was tested using the adhesive tensile method, referring to the national standards GB/T 8642–2002 and GB/T 23101.4–2008. First, a modified acrylate adhesive was uniformly applied to the surfaces of two mating parts, followed by careful positioning and fixing operations to ensure that the coating surface is perpendicular to the direction of the tensile force. After curing 24 h curing at room temperature, a tensile force was then applied at a constant speed of (1.0 ± 0.01) mm/min via a UTM5504X‐30KN universal electronic testing machine. Before the coating and substrate were totally separated, the maximum applied load and loading process was recorded.

### Mechanical Test

The size of three‐point bending samples was 55 mm × 10 mm × 4 mm (in accordance with Q/GB95‐92 standard) and the span was 40 mm. The test samples were loaded at a speed of 0.5 mm/min, and each test was repeated three times.

### Oxidation Test

The oxidation test under static air was widely used to evaluate the oxidation resistance of C_f_/C composites ranging from 800—1700 °C.^[^ ^47^, ^48^ ^]^ Herein, the β‐SiC and α‐SiC coated C_f_/C specimens were exposed to air flowing by natural convection at 1500 °C to evaluate their oxidation resistance using a muffle furnace (KSL‐1700X‐S). The muffle furnace was pre‐heated to 1500 °C at a heating rate of 5 °C min^−1^, and the coated samples in several Al_2_O_3_ crucibles were directly transferred into the high‐temperature chamber. The coated samples were taken out every 12–24 h and rapidly cooled down to room temperature in the air. The oxidation test contained several “Transfer to‐Fetch out” thermal cycles, and each cycle included three steps: (i) rapidly transfer the coated samples to high‐temperature furnace (1500 °C); (ii) keep the samples for a period at 1500 °C for anti‐oxidation evaluation; (iii) rapidly fetch the samples out and keep them cool to room temperature. Before oxidation test, all experimental alumina crucibles were placed in a heating furnace at 1500 °C and treated for 5 h to eliminate possible impurities. The oxidation performance was verified at multiple samples and evaluated at the average value. The mass loss (M%) of coated specimens was reckoned from the following:

(1) $$M\%=\frac{m_{0}-m_{t}}{m_{0}}\times 100\%$$

where *m*_0_ represents the initial mass of the coated specimens. *m_t_* represented the mass of coated specimens after *t* h oxidation.

### Molecular Dynamic Simulation

LAMMPS was used to analyze the oxygen diffusion and crack propagation behaviors in SiO_2_ film and β‐SiC coating. (i) For crack propagation process of SiO_2_/β‐SiC models with or without defects under tensile stress: The models in the size of 44 × 44 × 132 Å^3^ were pre‐treated at 300 K in NPT ensemble for 1000 steps to fully release internal stress. After relaxation, the models were put in NVT ensemble and stretched at a rate of 1 Å ps^−1^ for 20,000 steps along Z or X directions. The interatomic interactions within SiC and SiO_2_ were calculated using the Tersoff potential, while the interatomic interactions between C and O were calculated using the LJ potential. (ii) For amorphous SiO_2_ or mixed SiO_2_ under tensile stress, pre‐cracks (4 × 10 Å^2^) were introduced into the models in the size of 60 × 60 × 70 Å^3^ and pre‐treated at 300 K in NPT ensemble for 1000 steps to fully release internal stress. After relaxation, the models were put in NVT ensemble and stretched at a strain rate of 0.05 ps^−1^ based on the Tersoff potential. (iii) For three‐point bending process, a β‐SiC/graphene composite system was used, where the sample domain dimensions were 250 × 50 × 70 Å^3^, including the β‐SiC (50 Å) and the underlying graphene (20 Å) in the Z direction. After energy minimization, the system was relaxed at 300 K in the NVT ensemble with the boundary atoms fixed, followed by a spherical indenter (radius of 20 Å) entering into β‐SiC at a speed of 1 Å ps^−1^ along Z direction. (iv) For oxygen diffusion process: The calculation models comprised amorphous, cristobalite and mixed supercells with 100 oxygen atoms. The box size was set as 44 × 44 × 44 Å^3^ and the interactions between the atoms were described using Tersoff potentials. The models were pre‐relaxed in NPT ensemble to fully account for thermal expansion effects, followed by a 10 ns diffusion simulation in NVT ensemble. During the simulation, the mean square displacement (MSD) of the O atoms was output every 1000 steps (1 ps) to analyze the diffusion behavior of the oxygen atoms. The Einstein relation was employed to calculate the oxygen diffusion coefficients (D) in amorphous and mixed SiO_2_, which is expressed as follows:

(2) $$D=\frac{1}{2N}\frac{MSD}{t}$$

where *N* = 3 is the dimension of the system, *t* is the time of diffusion, and *MSD* is the mean square displacement.

## Conflict of Interest

The authors declare no conflict of interest.

## Supporting information



Supporting Information

## Data Availability

The data that support the findings of this study are available from the corresponding author upon reasonable request.
