# Supplementary material for: A Multi‐Step Deposition Strategy for β‐SiC Coatings on Cf/C Composites: Achieving Breakthrough Oxidation Resistance and Mechanical Properties
Source: Adv Sci (Weinh). 2025 Nov 18;13(7):e17256. doi: 10.1002/advs.202517256 (PMC12866845; doi:10.1002/advs.202517256)
Supplement: Supplementary file 1 — Supporting Information [file ADVS-13-e17256-s001.docx]

**Supplementary materials**

A Multi-step Deposition Strategy for β-SiC Coatings on C_f_/C Composites: Achieving Breakthrough Oxidation Resistance and Mechanical Properties

Dou Hu^†1,2^, Bing Liu^†,1^, Xiaoxuan Li^†,1^, Jia Sun^1^, Tao Li^*,3^, Yang Xu^*,2^, Hejun Li^1^, Qiangang Fu^*,1^

*1 Shaanxi Key Laboratory of Fiber Reinforced Light-Weight Composites, Northwestern Polytechnical University, Xi’an, Shaanxi, 710072, P.R. China*

*2 Department of Data and Systems Engineering, The University of Hong Kong, Hong Kong, P.R. China*

*3 Henan Key Laboratory of High Performance Carbon Fiber Reinforced Composites, Institute of Carbon Matrix Composites, Henan Academy of Sciences, Zhengzhou 450046, P.R. China*


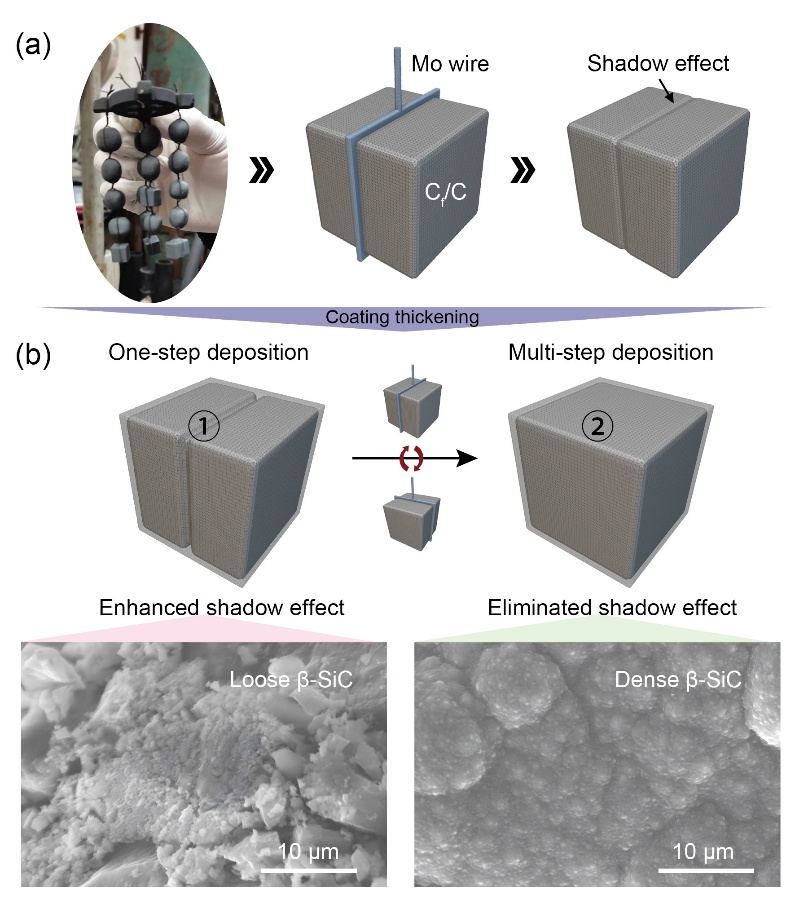


**Figure S1** Multi-step deposition strategy to eliminate the shadow effect while obtaining dense and thick β-SiC coatings. (a) Schematic diagram to reveal the formation of shadow effect during one-step deposition. (b) Effects of one-step and multi-step deposition strategies on shadow effect. By depositing in small amounts and multiple times without use of nucleating agents, the shadow effect of Mo wires can be reduced in thick CVD coating, thus avoiding porous coating structure and locally obvious defects.

Table S1 Summary of SiC-based anti-oxidation coatings for C/C composites

| Coating  systems | Design strategy | T (°C) | Atmosphere | Oxidation time (h) | Mass loss (%) | Ref. |
| --- | --- | --- | --- | --- | --- | --- |
| β-SiC  (1000~1500°C) | **Multi-step deposition** | **1500** | **Air** | **659** | **0.8** | **This work** |
|  | Single step | 1500 | Air | 6 | 18 | [24] |
|  | Single step | 1500 | Air | 10 | 5.0 | [26] |
|  | SiC_nw_ pre-network | 1500 | Air | 17 | 0.75 | [25] |
|  | [SiC/PyC]_n_ | 1500 | Air | 10 | 1.3 | [26] |
|  | [SiC/Ti_3_AlC_2_] | 1500 | Air | 30 | 0.64 | [30] |
|  | Single step | 1500 | Air | 4 | 32 | [31] |
| α-SiC dominated  (~2100°C) | PyC texture | 1500 | Air | 72 | 8.5 | [32] |
|  | Al, B additives | 1500 | Air | 12 | -0.3 | [33] |
|  | PyC layer | 1500 | Air | 49.5 | 1.1 | [34] |
|  | Al_2_O_3_ additive | 1500 | Air | 27 | 1.4 | [11] |
|  | SiC nanoparticle | 1500 | Air | 215 | 0.26 | [35] |
|  | ZrB_2_-SiC additive | 1500 | Air | 236 | 0.35 | [36] |
|  | ZrB_2_-SiC-Lu_2_O_3_ additive | 1500 | Air | 836 | -0.6 | [36] |
|  | MoSi_2_ pre-network | 1500 | Air | 581 | 1.83 | [37] |
|  | HfSi_2_-HfB_2_-SiC pre-network | 1500 | Air | 744 | -1.3 | [38] |
| Multiphase SiC  (β+α, 1600~1900°C) | SiC pre-network | 1500 | Air | 500 | -0.2 | [39] |
|  | ZrB_2_-MoSi_2_ | 1500 | Air | 62 | 0.28 | [40] |
|  | SiC pre-network | 1500 | Air | 110 | 0.4 | [41] |


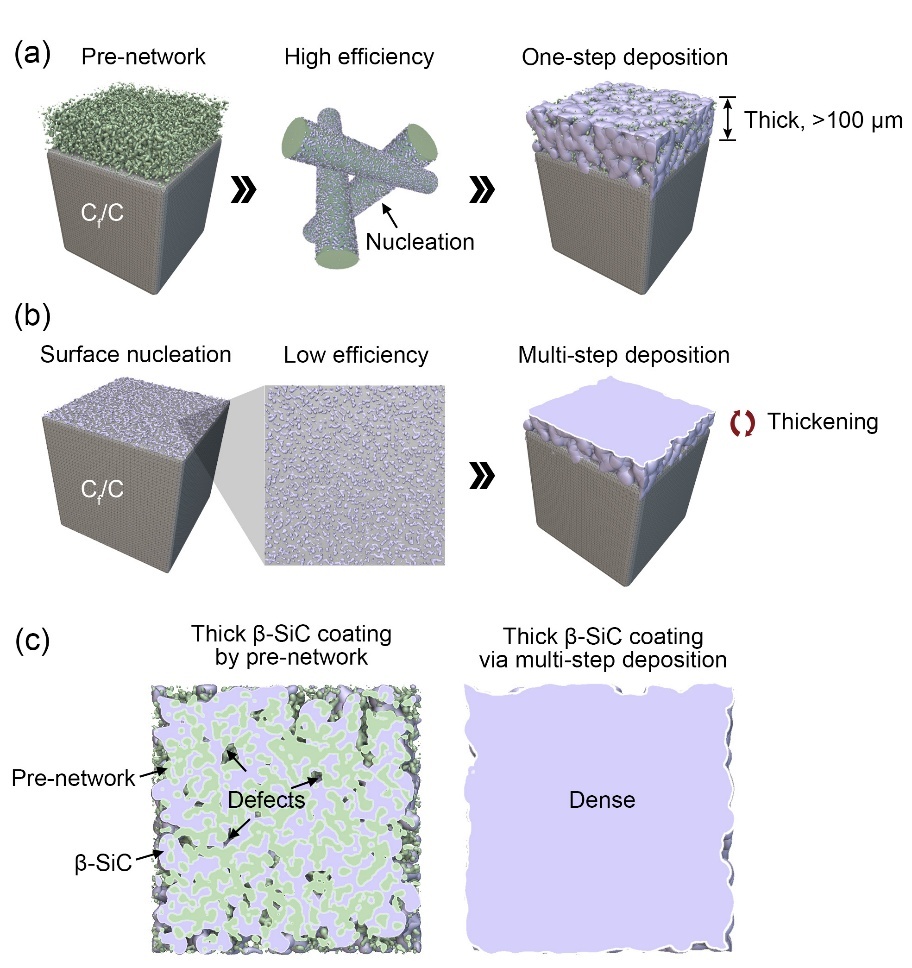


Figure S2 Strategy comparison between pre-network and multi-step deposition to construct thick coating. (a) Pre-network strategy by increased nucleation points to enhance coating deposition efficiency, thus improving the stability of coating/substrate interface. (b) Multi-step deposition strategy by multiple cycles to obtain thick coating, acquiring the balance between stability of coating/substrate interface and coating internal density. (c) Typical characteristics between pre-network reinforced β-SiC and multi-step β-SiC.


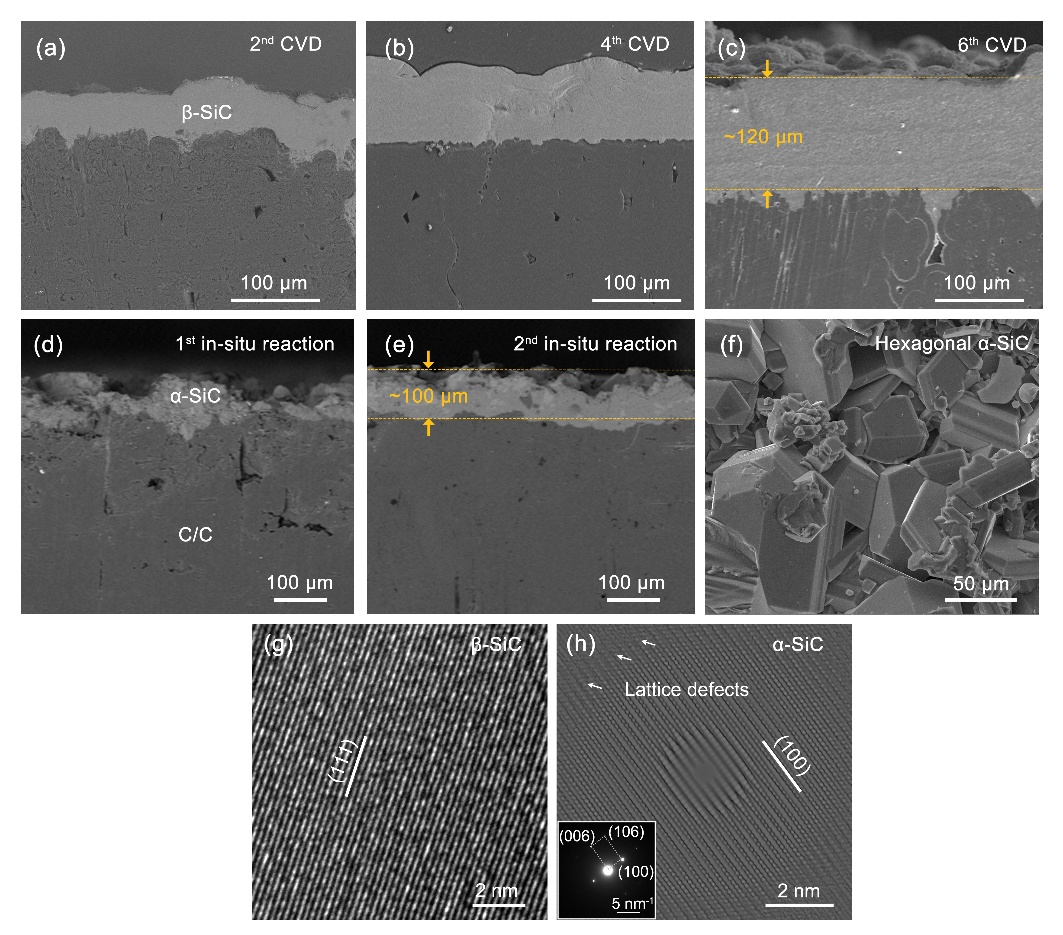


**Figure S3** SEM and TEM images of deposited β-SiC and α-SiC coatings. (a-c) Cross-section pictures of β-SiC coatings after two, four and six chemical vapor deposition cycles. (d-f) Cross-section and surface images of α-SiC coatings after one and two in-situ reaction cycles. HRTEM images of β-SiC (g) and α-SiC (h) coatings.


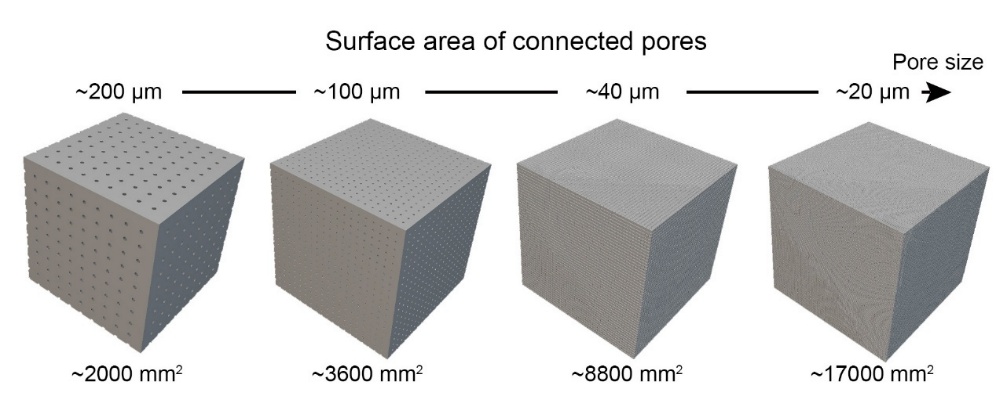


Figure S4 Surface area of connected pores with the decrease of pore size at a total porosity of 10%. Herein, the open pores are simplified as connected column pores, and the cubic region is set as 10×10×10 mm^3^. Their surface area significantly increases with pore size declining. Compared to completely dense structure, 10% porosity will bring over 170 times increase in surface area at the average pore size of 20 μm.


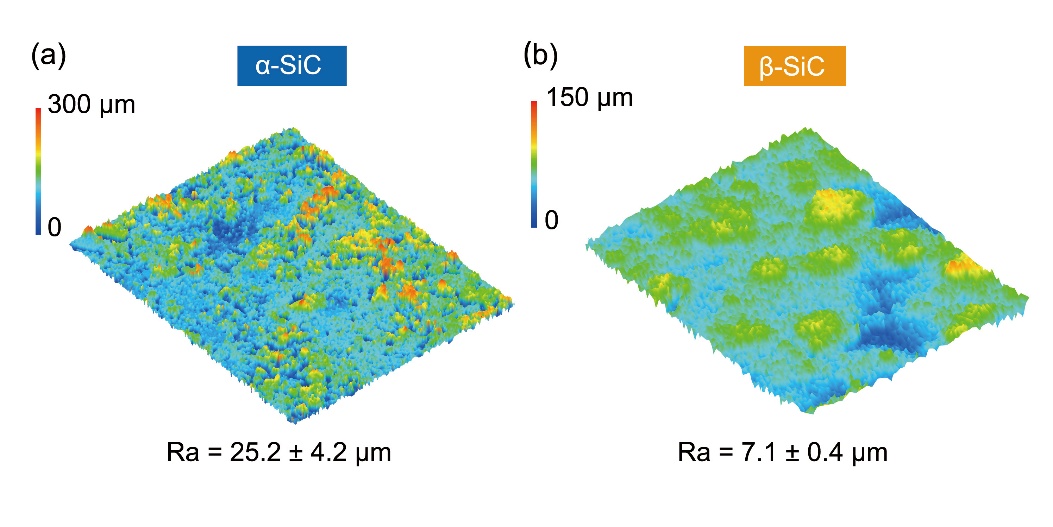


**Figure S5** Surface roughness of (a) α-SiC and (b) β-SiC coated C_f_/C composites. The β-SiC coatings prepared by CVD exhibit much higher surface quality, the average roughness of which is no more than 1/3 of in-situ reacted α-SiC coatings.


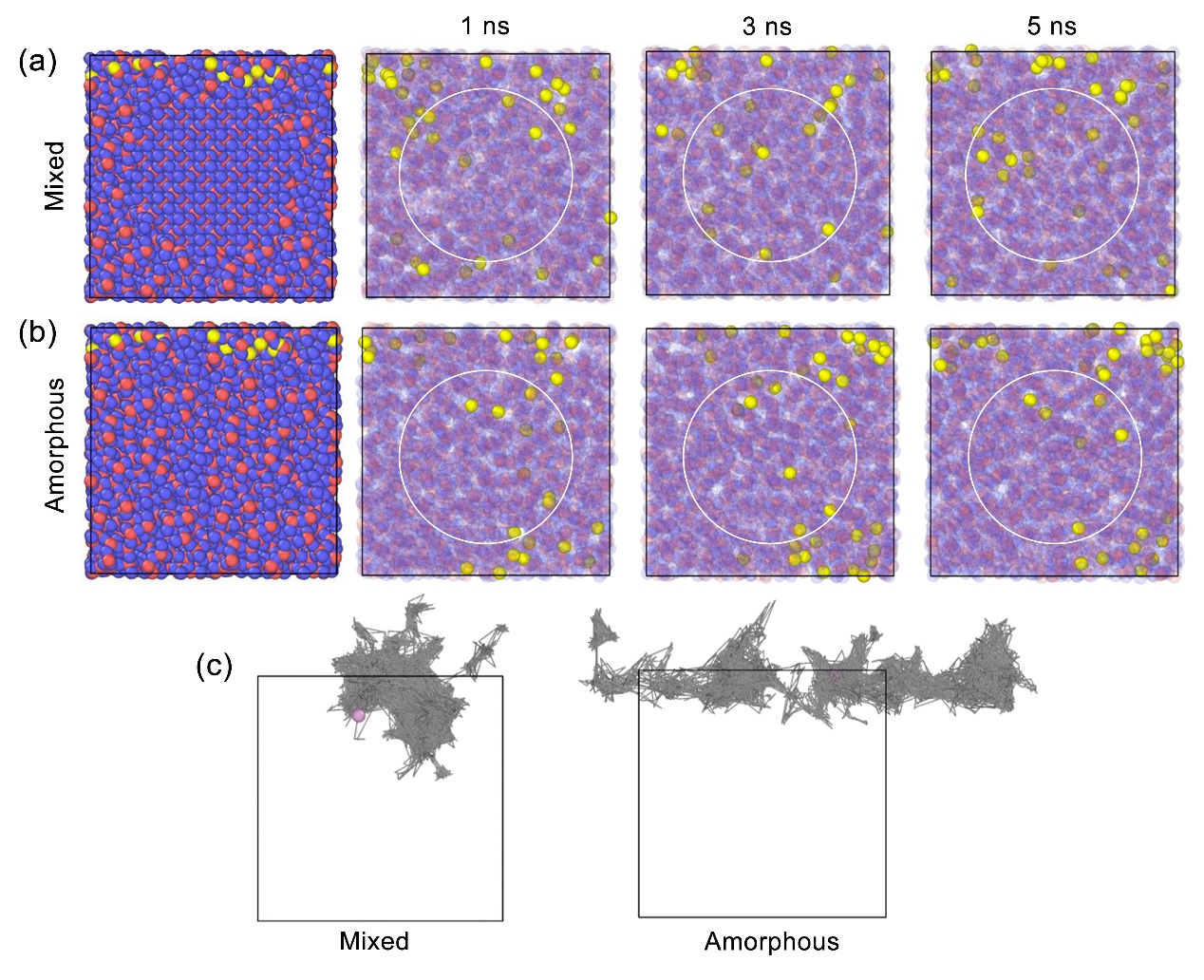


**Figure S6** Dynamic O_2_ diffusion process at 1500°C in the (a) mixed and (b) amorphous SiO_2_ films. (c) The movement trajectory of single oxygen atom. The crystal area of mixed SiO_2_ film shows the inhibition effect on O_2_ molecules.


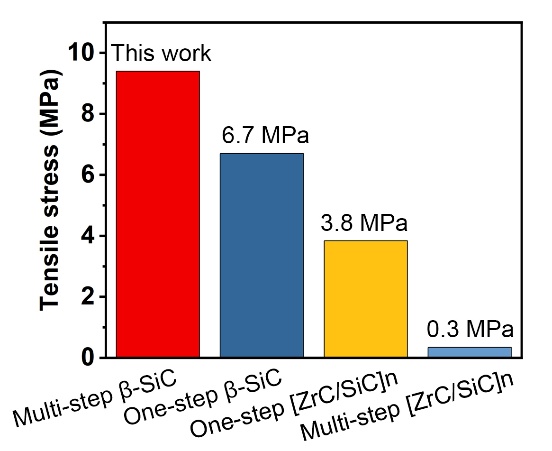


Figure S7 Comparison of adhesion strength between our strategy and other one-step or multi-step deposited coatings [23, 26], which suggests the strong interface bonding in this work.


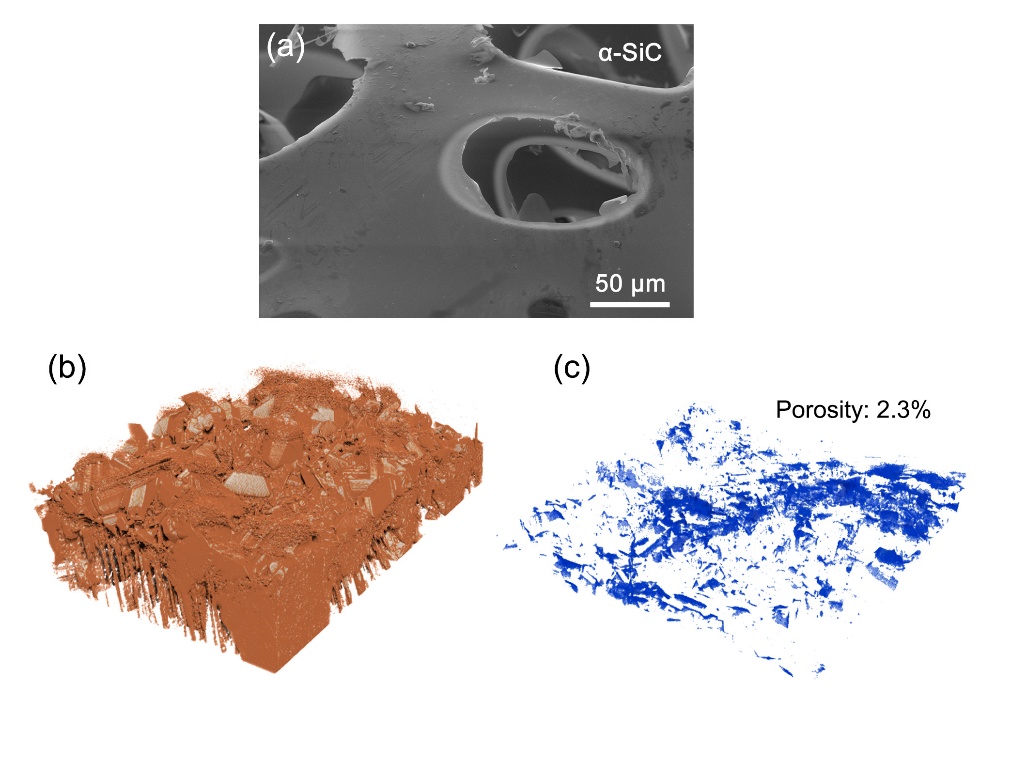


**Figure S8** The oxidation behavior and crack resistance of α-SiC coating by liquid Si in-situ reaction. (a) Surface SEM image after 27 h oxidation at 1500°C. (b) Micro-CT image of surface α-SiC multi-grains and (c) porosity analysis on α-SiC coating.


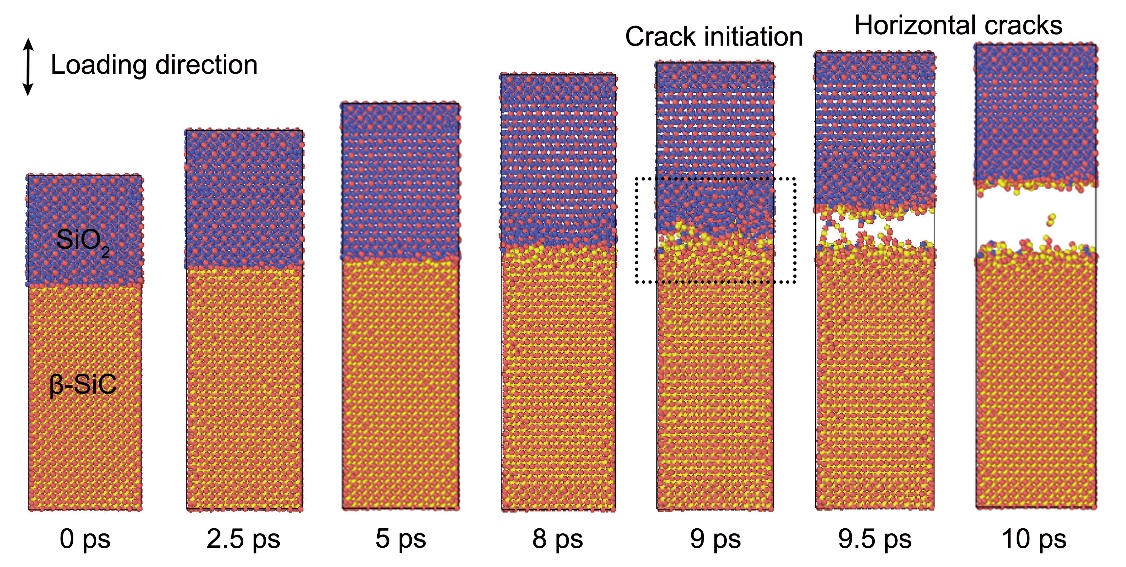


**Figure S9** Crack initiation and propagation process at the interface SiO_2_ and SiC.


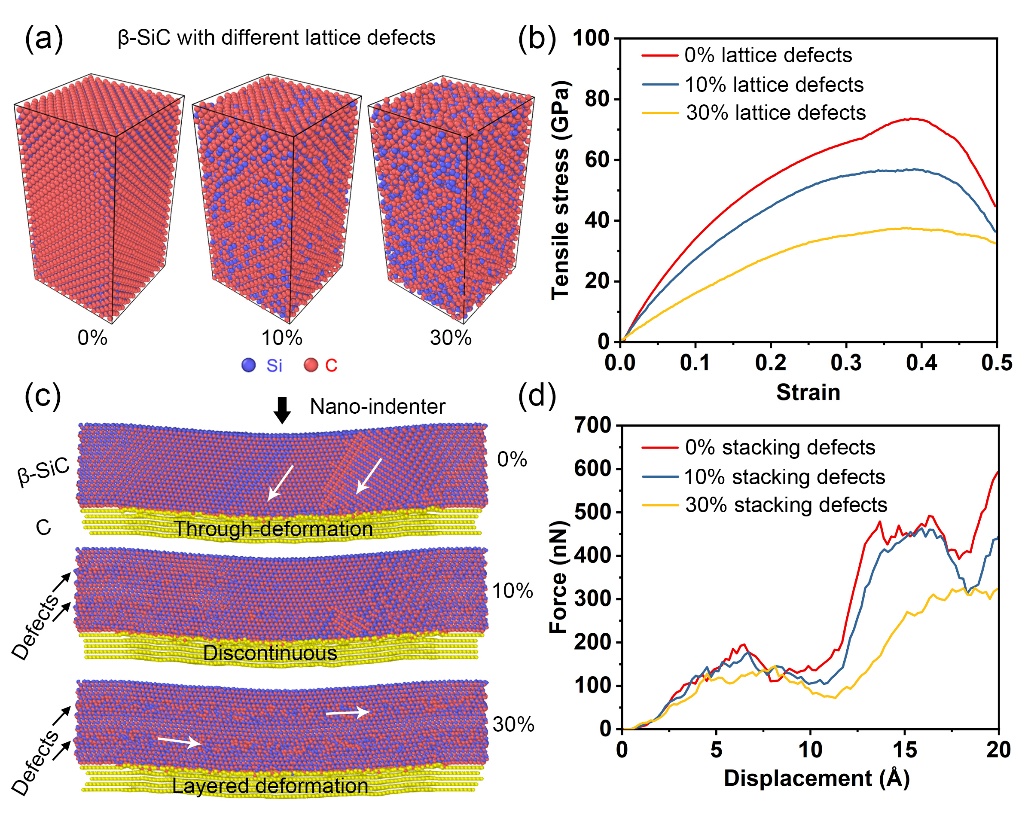


Figure S10 Deformation behavior of β-SiC coatings with different stacking defects. (a) Lattice structures and (b) their tensile stress-strain curves with defect content increasing. When the amount of lattice defects exceeds 10%, the initial lattice structure shows a slight deconstruction and its tensile stress decreases by 22%. (c) Deformation behavior of β-SiC coatings with different interface stacking defects under nano-indentation process. A small number of stacking defects at the interface will be helpful for the deformation discontinuity to inhibit penetrating cracks. (d) Force-displacement curves under different stacking defect contents (0%, 10% and 30%). Despite up to 10% stacking defects, the fracture force is almost unchanged.


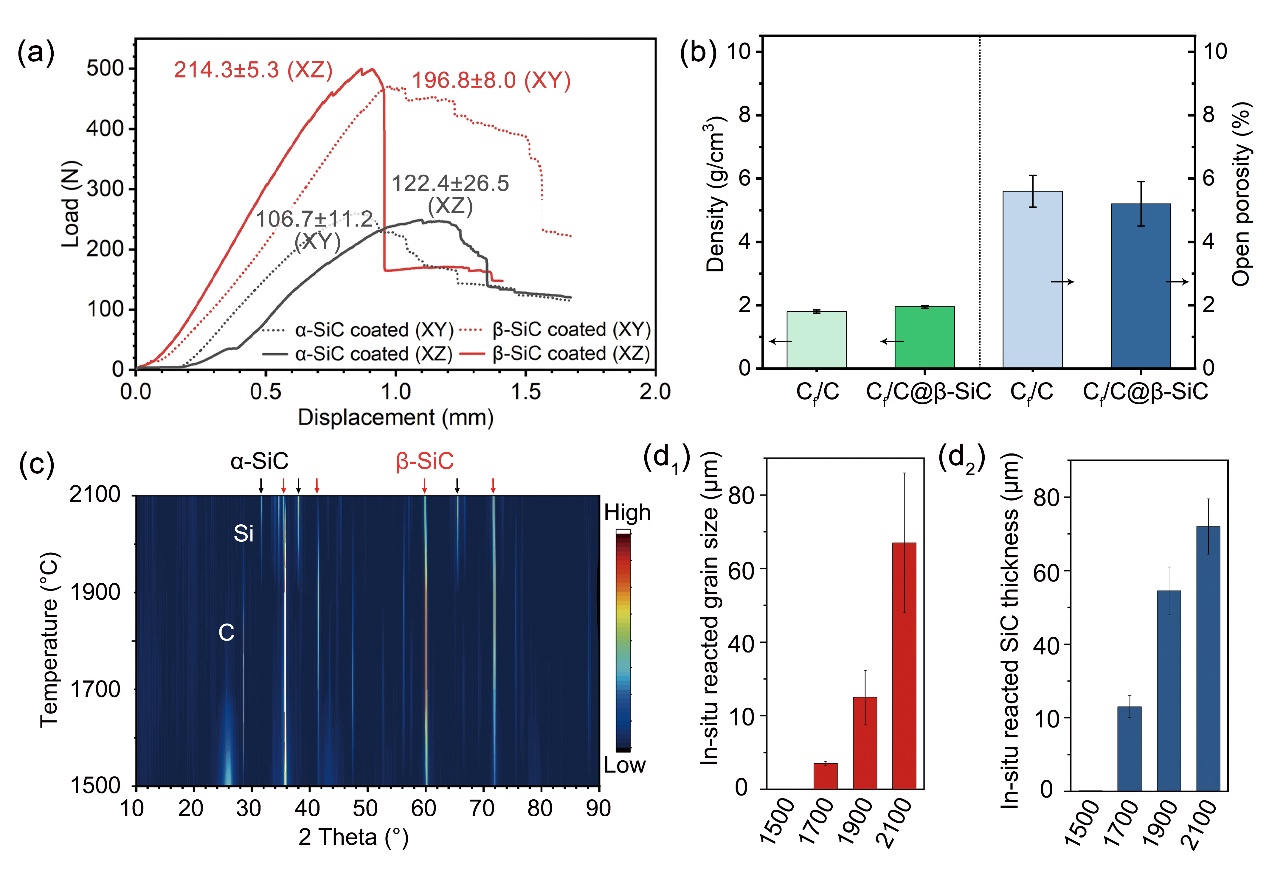


**Figure S11** (a) Load-displacement curves of α-SiC and β-SiC coated C_f_/C composites along XY and XZ planes. This indicates the enhancement efficacy of β-SiC coating despite anisotropic C_f_/C substrate. (b) Total density and open porosity of β-SiC coated C_f_/C composites, which demonstrates its hindering effects on surface defects from original C_f_/C composites. (c) XRD patterns of in-situ reacted SiC layers on carbon fibers after heat treatment at different temperatures, which reflects the aggravated damage above 1700°C. Evolution of in-situ reacted SiC (d_1_) grain size and (d_2_) layer thickness, suggesting that the aggravated damage above 1700°C results from increased carbon fiber consumption.
